# The Effect of Economic, Physical, and Psychological Abuse on Mental Health: A Population-Based Study of Women in the Philippines

**DOI:** 10.1155/2014/852317

**Published:** 2014-11-27

**Authors:** Diddy Antai, Ayo Oke, Patrick Braithwaite, Gerald Bryan Lopez

**Affiliations:** ^1^Centre for Public Health Research, School of Health Sciences, City University London, Northampton Square, London EC1V 0HB, UK; ^2^Division of Global Health & Inequalities, The Angels Trust-Nigeria, Abuja, Nigeria

## Abstract

*Background*. The comparative effect of economic abuse and other forms of abuse in predicting depression and other mental health disorders has not been previously investigated despite its relevance for mental illness prevention. *Objective*. To determine the differential association of economic abuse on psychological distress and suicide attempts. *Study Design*. We used cross-sectional data from women aged 15–49 years in the 2008 Philippines Demographic and Health Surveys (PDHS) (*N* = 9,316). *Results*. Adjusting for sociodemographic confounders revealed positive associations between economic, physical, or psychological abuse and suicide attempts and psychological distress. Psychological and economic abuse were the strongest predictors of suicide attempts and psychological distress, respectively. Economic abuse was also negatively associated with psychological distress. Comorbidity with one mental health disorder greatly increased the odds of reporting the other mental health disorder. *Conclusion*. Overall, the results elucidate the differential effects of these forms of abuse on women's mental health.

## 1. Introduction

Is economic abuse more predictive of depression and other mental health disorders than other forms of abuse? This association has not been adequately investigated in spite its relevance for mental illness prevention [1]. Intimate partner violence (IPV) is one of the most common forms of violence against women that is performed by a husband or an intimate male partner [2]. It is a serious public health problem associated with physical [3, 4], reproductive [2, 5], and mental health [6–8] consequences. The global dimensions of IPV are alarming, with about 15–71% of women reporting experiencing IPV over their lifetime [9]. Various forms of abuse generally coexist within the same relationship; however, reported estimates are sensitive to definitions used, the manner in which questions are asked, the degree of privacy during interviews, and the gender norms of the population [10]. IPV is commonly conceptualized as a pattern of coercive behaviours in a relationship whereby one partner uses tactics of power and control over the other partner over a period of time [11]. Despite the abundant literature on the different types of abuse, very few have focused on economic abuse, with scholars stressing the need to include economic abuse as a form of IPV, given the nature of behaviours such as employment sabotage, economic control, and economic exploitation [12–14].

### 1.1. Economic, Physical, and Psychological Abuse and Women's Mental Health

Economic abuse, in addition to physical, sexual, and psychological abuse, is a common tactic of control in IPV [15] that is as common as physical and psychological abuse. Defined as the “control of a woman's ability to acquire, use, and maintain economic resources, thus threatening her economic security and potential for self-sufficiency” [12], it is a coercive behaviour that makes the victim economically dependent on her partner and at greater risk of continued abuse [16]. By controlling women's ability to acquire, use, and maintain economic resources, economic abuse threatens victims' economic security and ability to achieve economic independence [17]. To establish a state of economic dependence, an abuser might take “control” of a woman's resources by* preventing her from obtaining and maintaining employment outside the home* [18, 19], causing her job absence or loss by showing up at her work place [20];* preventing women's use of* existing* resources* by controlling resource distribution and use [18, 19, 21], denying them access to joint bank accounts or financial information [18, 19]; and* exploiting a woman's resources* by stealing her money, creating costs, and generating debt, thereby depleting her economic resources [19, 21]. Economic abuse can lead to poverty, which, in turn, is a risk factor for further economic abuse [22]. In addition to creating financial dependence, economic abuse creates a “hostile” environment where the abused woman is continually psychological distressed and anxious about material or financial issues. As the victim's financial dependence on the abuser increases, leaving the relationship becomes more difficult. Studies have found this stress to be linked with depression, anxiety, and parenting problems [1, 23].

Physical abuse in intimate relationships is often accompanied by psychological abuse and in one-third to over one-half of cases by sexual abuse [24]. Evidence suggests that psychological abuse may have equally deleterious effects on women's mental health, if not more so, than physical abuse [8]. However, frequent cooccurrence of psychological abuse with physical violence masks the independent effects of psychological abuse on mental health [25]. These studies however focused on physical, sexual, and/or psychological abuse, with the effect of economic abuse either being sparsely investigated or its mental health effects being adequately investigated. This study therefore seeks to address this gap by using self-reports of abuse in a population-based sample of women to examine the unique effects of specific types of abuse (i.e., physical, psychological, or economic) on such maternal mental health outcomes as psychological distress and suicide attempts.

## 2. Background

### 2.1. Suicide Attempt

Suicide is a global public health problem [26] and major contributor to the global burden of disease [27]. Globally, intentional self-inflicted injuries constitute a substantial health burden by being the 4th leading cause of death and 7th leading cause of disability-adjusted life years (DALYs) for women aged 15–44 in 2005 [28]. Despite abundant epidemiological research on the prevalence of suicide in high-income countries, limited data exists in low- and middle-income countries [29]. Although the causes of suicide are multiple and interact in complex ways, mental health problems, particularly depressive disorders, are some of the best-known risk factors associated with suicide ideation, suicide attempts, and suicide mortality [30]. There is increasing interest in understanding how psychological trauma may affect suicidal behavior, with studies indicating a strong positive association between history of IPV and suicidal behaviours among women [31] and the risk for suicidal ideation or attempts increasing with IPV severity.

### 2.2. Psychological Distress

The most prevalent mental health consequences reported in the past two decades have been depression, posttraumatic stress disorder (PTSD), and anxiety [3]. Despite psychological distress being commonly used as an indicator of the mental health status of a population in public health, it is still a relatively vague concept. Psychological distress is defined as “a state of emotional suffering characterized by undifferentiated combinations of symptoms ranging from depression and general anxiety to personality traits, functional disabilities, and behavioural problems” [32]. Associated with somatic symptoms such as insomnia, headaches, lack of energy often varies across cultures, psychological distress has been variedly described as an emotional disturbance that may impact on the social functioning and day-to-day living of individuals [33]. It is a medical concern, especially when accompanied by other symptoms, that, together, satisfies the diagnostic criteria for a psychiatric disorder [34]. Serious psychological distress is reported to be highest (15.4%) among women exposed to lifetime physical and sexual IPV compared to those not exposed to among those with no lifetime experience of IPV [35].

### 2.3. Theoretical Framework Linking Physical, Psychological, and Economic Abuse to Psychosocial Stress and Suicide Attempts

Evidence indicates a complex relationship between IPV and depression, suicide attempts, and other common mental disorders, which tends to be context-specific. There is a strong positive association between IPV and suicidal behaviours in women [31] and psychological distress in both high- and lower-income settings [36]. Traumatic stress causes fear and isolation, which in turn might lead to depression and suicidal behaviour, thus becoming the main mechanism by which IPV might cause depression and suicide attempts [37]. This relationship is reported to be bidirectional [37] in that women with severe mental health difficulties are more likely to experience violent victimisation [38]. Although it is clear that violence must precede completed suicides, most studies on violence and suicide actually measure suicide attempts, which could precede violent experiences [38]. Evidence from longitudinal studies also shows that violent experiences may lead to greater psychological distress [39], which may also interfere with economic wellbeing. The link between economic abuse and depression has not been well identified. Coercive control theory portrays an abuser as one who attempts to gain power and control insidiously over his/her partner by using a variety of control tactics [15], including violence with the aim of coercing her to become economically dependent on him alone [1]. It is plausible that having a partner control access to financial resources or preventing economic independence through work or school adversely affects a woman's mental health, especially when such experience is long term [13]. Although money may not be a guarantor of mental wellbeing, nor does its lack necessarily lead to mental illness, poverty could be both a determinant and a consequence of poor mental health [40].

## 3. The Current Study

Using a nationally representative data from the Philippines, this study builds on the only other available study in three key ways. First, the present study is novel in that it determines the differential association of economic, physical, and psychological abuse on psychological distress and suicide attempts; no previous studies have previously examined this relationship. Second, we consider the effect of controlling for sociodemographic characteristics, and third, we account for the effects of cooccurring mental disorders, by performing sensitivity analyses that explored the potential role of comorbidity with the other mental health outcome to assess whether the added experience with the other mental health outcome led to higher levels of the mental health outcome being examined. Finally, we provide empirical evidence on the relationship between forms of abuse and mental health consequences originating from a low- and middle-income context, given that previous evidence have originated from high-income contexts, which do not necessarily reflect cultural differences within low-middle-income contexts such as the Philippines where the cultural context is important in determining power dynamics in IPV and shaping how relationship power is related to the risk of mental health consequences of IPV [41].

## 4. Methods

### 4.1. Data

Data for this study was derived from the 2008 Philippines Demographic and Health Surveys (PDHS). The PDHS is part of a large survey progamme carried out in over 70 low- and middle-income countries that measure and evaluate key population, health, and socioeconomic and anthropometric indicators within the surveyed countries, with emphasis on maternal and child health [42]. To ensure standardization and comparability across countries and time, the DHS employs intense interviewer training, standardized data-processing guidelines, an identical core questionnaire, and instrument pretesting [43]. The PDHS are nationally representative cross-sectional household sample surveys conducted by face-to-face interviews of women aged 15–49 years in the homes of respondents between February 2001 and April 2003.

### 4.2. Sampling Plan

The 2008 PDHS was conducted as a multistage stratified clustered area probability sample of the Philippines household population [42], with a household response rate of 99 percent. Using a sampling frame from the most recent population census, a three-stage cluster sample design representing 17 administrative regions, a sample of 13,764 households (response rate of 99.3%) was randomly selected from 794 primary sampling.

Units (PSUs), from which an initial nationally representative sample of women (*N* = 13,594), were collected. From these women, information on topics relating to women's experiences of violence since age 15, violence during pregnancy, marital control, and intimate partner violence was collected from women (*N* = 9,316) selected using the Women's Safety Questionnaire/Module. Detailed descriptions of the study design and methods of data collection are accessible online in household survey reports [44].

### 4.3. Measures

#### 4.3.1. Outcomes

Two indicators of mental health were examined as the outcomes of interest.* Psychological distress* was measured as a variable derived from the women's response to the question “have you ever had depression, anxiety, anger, sleeplessness, irritable, confused, feeling of isolation because of husband's act?” Responses were presented as a dichotomous “yes” or “no” variable (yes = 1).

*Suicide attempt* was measured from responses to the question “have you ever attempted to commit suicide because of husband's act?” Responses were presented as a dichotomous “yes” or “no” variable (yes = 1).

#### 4.3.2. Exposures

Economic, psychological, and physical abuse were the main exposure variables.

*Economic abuse* was measured by four items “disallowed respondent to engage in legitimate work,” “controlled money or force her to work,” “destroyed personal property/pet or threaten to harm pet,” and “ever lost job/source of income because of husband.” These items were derived from responses to questions asked to respondents about whether their spouse had ever exhibited the behaviours in question. Responses were presented as dichotomous “yes” or “no” variables (yes = 1, no = 0). A composite binary variable constituting 2 or more items of economic abuse was not created as these composite variables did not meet satisfactory Cronbach's alpha level of .70.

*Physical abuse *was measured using seven items indicating lifetime experience of physical violence.

Using items from the Conflict Tactics Scale [45], respondents were asked if their current or most recent partner had done the following: (i) pushing, shaking, or throwing something at her; (ii) slapping her or twisting her arm; (iii) punching or hitting her with something harmful; (iv) kicking or dragging her; (v) strangling or burning her; (vi) threatening her with a weapon (e.g., gun or knife); and (vii) twisted her arm.

*Psychological abuse* was measured using two items indicating lifetime experience of psychological violence: (i) humiliating her in public; and (ii) threatening her or someone close to her.

There were five possible responses for each item of abusive act: “no,” “often,” “sometimes,” “not at all,” and “yes,” from which responses of “no” and “not at all” to any of the items were recoded to indicate the response “no” = 0, and responses of “yes,” “often,” and “sometimes” were recoded to indicate the response “yes” = 1. The seven items indicating lifetime experience of physical violence and the two items indicating lifetime experience of psychological violence were scaled additively to yield measures of internal validity of Cronbach's alpha (*α*) = of .90 for physical violence, and (*α*) = .80 for psychological violence.

#### 4.3.3. Sociodemographic Characteristics

Demographic variables were respondents' age group (15–19, 20–24, 25–29, 30–34, 35–39, 40–45, and 46–49 years), marital status (currently married and never married).

Socioeconomic status included educational level (no education, primary education, and secondary or higher education), current occupational status (employed, and unemployed), and place of residence (urban and rural). Other variables were (i) controlling behaviour, measured as a composite binary variable created from six questions regarding controlling acts by present or former husband/partner: jealous if she talks with other men, accuses her of unfaithfulness, does not permit her to meet her friends, tries to limit her contact with family, insists on knowing where she is, and does not trust her with money. Responses of “yes” to one or several of the six questions were considered as “yes” and coded 1, whilst responses of “no” to all the questions made up the response alternative “no” and coded 0. Cronbach's alpha was (*α*) = was .90 for controlling behaviour; and (ii) Justify wife beating, measured as a composite binary variable created from responses to five questions enquiring whether the respondent would justify abuse of a woman by her partner for such reasons as “when she goes out without telling him,” “neglects the children,” “argues with him,” “refuses to have sex with him,” and “burns the food.” The response alternative “yes” was defined as the women's response of “yes” to one or several of these attitude questions, coded “yes” = 1, and “no” was defined as responses of “no” to all the attitude questions, code “no” = 0. Cronbach's alpha (*α*) was .907.

## 5. Statistical Analysis

The analyses were performed in steps. First, cross-tabulations between outcome and exposure variables with significant levels determined by chi-square (*χ* ^2^) analyses and set at *P* < 0.05 were performed. Only significant variables from bivariate analyses with each outcome variable that fulfilled the criteria for being confounders were included in multivariate analyses [46]; this approach reduces the number of variables until the most parsimonious model describing the data is attained (which contains the important confounders). Second, we assessed the individual crude/bivariate associations between the outcomes (psychological distress, and suicide attempt) and the main exposure variables (economic, physical, and psychological abuse). Third, we assessed the adjusted/multivariate associations between outcomes and the main exposures, with the main exposure variables inserted into the analysis in a single block. Fourth, we assessed the adjusted/multivariate associations between the outcomes and the main exposures, including potential sociodemographic confounders. We also performed a couple of sensitivity analyses. First, depending on the outcome being analyzed, we included suicide attempt or psychological distress as exposure variables in Model 3 to assess for influence of comorbidity with the other mental health outcome. For example, in the analysis of suicide attempt as outcome variable, psychological distress was included as an exposure variable. All variables were entered into the multivariate logistic regression models in a single block to control for possible confounding between these variables. Results were expressed as odds ratio (OR) with their 95% confidence interval levels (95% CI). Statistical analyses were performed using PASW 19.

## 6. Results

### 6.1. Demographic and Socioeconomic Characteristics of the Study Sample

The higher the educational level, the higher the proportion of women who reported more suicide attempts. In contrast, the proportion of women who reported suicide attempts decreased with increasing wealth of the household. Employed and currently married women reported significantly more suicide attempts than unemployed and never/formerly married women, respectively. Rural women and women with controlling male partners reported significantly more suicide attempts than urban women and women who did not have controlling partners. Significantly more women with psychological distress were employed, rural residents and had male partners with controlling behaviours compared to their unemployed and urban counterparts, as well as those whose partner did not have male partners with controlling behaviours, respectively. The poorer the household, the higher the proportion of women who reported psychological distress. Women's age was nonsignificant in relation to suicide attempts and psychological distress (Table 1).

### 6.2. Outcome and Main Exposure Characteristics

The association between measures of economic, physical, and psychological abuse and suicide attempts and psychological distress was significant and positive with the exception of the association between one economic abuse measure (“not allowed to engage in legitimate work”) and psychological abuse, which was significant and negative (Table 2).

### 6.3. Association between Type of Abuse, Suicide Attempts, and Psychological Distress

As presented in Table 3, crude associations between outcomes and main exposure variables (Model 1) showed that economic, physical, and psychological abuse were significantly associated with increase of the likelihood of suicide attempts and psychological distress, with the exception of the economic abuse measure “not allowed to engage in legitimate work,” which significantly decreased the likelihood of psychological distress. The strongest crude association was between the economic abuse measure “spouse controlled money or forced her to work” and suicide attempts (crude odds ratios [crude OR] = 4.49; 95% confidence interval [CI] = 2.96–6.80) and psychological distress (crude OR = 2.95; 95% CI = 2.02–4.32) compared to those not exposed to this form of abuse, respectively. Adjusting for all the measures of economic, physical, and psychological abuse introduced into the model in a block (Model 2) largely attenuated the crude/bivariate associations, resulting in two economic abuse measures (“not allowed to engage in legitimate work” and “destroyed personal property/pet or threaten to harm pet”) becoming nonsignificant in association with suicide attempts. The strongest adjusted association was between the psychological abuse and suicide attempts (adjusted odds ratios [adjusted OR] = 2.19; 95% confidence interval [CI] = 1.54–3.11) and between the economic abuse measure “ever lost job/source of income because of husband” and psychological distress (adjusted OR = 2.16; 95% CI = 1.49–3.13). The likelihood of psychological distress in relation to the economic abuse measure “not allowed to engage in legitimate work” remained significantly lowered.

Adjusting for confounders (Model 3) further attenuated the outcomes-exposures associations, which remained relatively unchanged from the previous model. However, the association between the economic abuse measure “spouse controlled money or forced her to work” and psychological distress became nonsignificant. Psychological abuse (adjusted OR = 2.02; 95% CI = 1.42–2.88) was the strongest predictor of suicide attempts whilst the economic abuse measure “ever lost job/source of income because of husband” (adjusted OR = 2.16; 95% CI = 1.49–3.13) was the strongest predictor of psychological distress. The lower likelihood of psychological distress in relation to the economic abuse measure “not allowed to engage in legitimate work” remained unchanged from the previous model. Model 3 also showed that being unemployed decreased the odds of reporting suicide attempts (adjusted OR = 0.63; 95% CI = 0.46–0.86) and psychological distress (adjusted OR = 0.81; 95% CI = 0.68–0.96) compared to being employed. The odds of reporting suicide attempt were significantly higher for women in the 2nd poorest quintile (OR = 2.15; 95% CI = 1.03–4.48) and middle quintile (OR = 2.21; 95% CI = 1.06–4.62) compared to their counterparts in the richest wealth quintile. Wealth index was not significantly associated with psychological distress. Having a controlling male partner increased the odds of women reporting suicide attempts (OR = 1.66; 95% CI = 1.11–2.49) and psychological distress (OR = 1.43; 95% CI = 1.19–1.72) compared to women without a controlling partner. Rural residence (OR = 1.32; 95% CI = 1.09–1.58) increased the women's likelihood of reporting psychological distress.

### 6.4. Sensitivity Analyses Confounding by Comorbid Mental Illness

We adjusted for comorbidity with the other outcome variable, depending on the outcome being analyzed. Comorbidity with psychological distress increased the odds of reporting suicide attempts (OR = 9.64; 95% CI = 5.82–15.96). In addition, comorbidity with suicide attempts also greatly increased the odds of reporting psychological distress (OR = 9.57; 95% CI = 5.79–15.81).

## 7. Discussion

The primary aim of this study was to investigate the differential effects of economic, physical, and psychological abuse in the prediction of suicide attempts and psychological distress. Key findings from this study provide better understanding of the relationship between these types of abuse and women's mental health outcomes. First, after controlling for sociodemographic confounders, women who experienced economic abuse (“spouse controlled money or forced her to work” and “ever lost job/source of income because of husband”) or physical or psychological abuse were more likely to carry out suicide attempts. Additionally, women who experienced economic (“destroyed personal property/pet or threaten to harm pet” and “ever lost job/source of income because of husband”), physical, or psychological abuse were more likely to experience psychological distress. In contrast, women who experienced the economic abuse measure “not allowed to engage in legitimate work” were less likely to experience psychological distress. To the best of our knowledge, findings linking economic abuse with suicide attempts and psychological distress have not been previously identified. It is plausible that having an abusive partner that makes all her financial decisions and maintains complete control over her money and other economic resources reduces the victim's ability to acquire, use, and sustain economic resources. This in turn renders the victim economically dependent on her partner and creates a stressful home environment where the victim is constantly anxious about financial or material issues; stress of this type has been shown to be associated with depression and anxiety [1, 23].

The finding that the economic abuse indicator (“not allowed to engage in legitimate work”) lowered the odds of reporting psychological distress is an interesting finding that is not easily explained. Given that what constitutes violence or abuse may vary across social contexts, it is plausible that respondents may not have perceived or understood the statement “not allowed to engage in legitimate work” as a form of economic abuse. It is also plausible that work may be a source of psychological distress for some of these women, especially psychological distress resulting from employers or working conditions, and the added demands of family responsibilities. Further investigations are needed to obtain a better understanding of the indicators of economic abuse, as they may require different types of screening and/or intervention methods. Economic abuse (“ever lost job/source of income because of husband”) was also more predictive of psychological distress than either physical or psychological abuse. This is also a novel finding, with plausible explanations being that loss or depletion of key resources (e.g., employment) necessary to deal with the trauma of abuse and high levels of relationship dissatisfaction may lead to increased psychological distress [47]. Implications of this finding, aside from the evidence that unemployment constitutes a serious threat to mental health, include the need for healthcare personnel to formulate psychological interventions to include screening for mental disorders for unemployed abused women, as evidenced by positive effects of such measures in prior studies [47].

Second, our finding that women who experienced physical or psychological abuse were more likely to attempt suicides and experience psychological distress, with psychological abuse being more predictive of suicide attempts, agrees with previous studies in a range of low- and middle-income countries [31, 37, 48]. Our finding is also consistent with those from other studies indicating positive association between the lifetime experience of abuse and psychological distress [49]. Similar to the finding in other studies [8, 50], this present study found that psychological abuse was a stronger predictor of suicide attempts than physical abuse, after adjusting for explanatory factors; however, this finding contrasts with that in another study [51]. It is plausible that the stress and experience of physical or psychological abuse may manifest as internalizing disorders such as depression, anxiety, or PTSD [52], as well as suicidal ideation. Further qualitative research is needed to fully understand the link between experience of severe abuse and suicidal behaviors, paying special attention to potential mediating effects of mental disorders, especially if these patients do not present with overt psychiatric disorders or attempt health. The clinical implication for this is the tendency of healthcare/emergency physicians not to screen abused patients for mental health suicides. In general, all victims of abuse should receive a mental health evaluation given the high psychiatric burden among survivors of abuse. This would increase recognition of depressive symptoms, especially in patients at risk for suicide attempts, given that suicide attempt is a well-recognized risk factor for completed suicide [53], and would enhance the chances of abused women of getting psychiatric treatment.

Third, several sociodemographic variables that influence the associations between intimate partner abuse, suicide attempts, and psychological distress were identified. Unemployment was inversely associated with suicide attempts and psychological distress contrary to the commonly documented finding of unemployment as a risk factor for suicidal behaviour [54]. Our finding is an interesting one that warrants further explanation: one of three possible explanations being that the association may be* noncausal* (wholly or partly spurious) due to confounding or selection by factors that predict or are antecedent to unemployment but were related to suicide attempt risk such as lack of formal educational qualifications, childhood sexual abuse, and poor parental marital relationship [55]. Further studies that adjust for antecedent childhood and family factors as well as psychiatric morbidity are needed, as these may show that unemployment may not be significantly related to risks of suicide attempt, as seen in other studies. One other plausible explanation is that high rates of unemployment in the Philippines may be reflective of individuals with psychiatric morbidity. In this case, the association between unemployment and suicide attempt is likely to be noncausal and is due to unemployment being symptomatic of high risk individuals who are characterized by a combination of psychosocial, family, and educational adversity and/or psychiatric morbidity. In addition, mental illness is a likely intermediatory factor between unemployment and suicide [56].

Our finding that respondents in the middle and poor wealth categories (2nd poorest and middle wealth quintiles) were more likely to attempt suicides compared with those in the highest wealth category corroborates findings from other studies [57]. The present study supports the social causation theory that adversity, stress, and reduced capacity to cope related to low-income increases the risk of mental disorder [58]. Mechanisms such as violence and a limited capacity to acquire healthcare for physical health problems might increase the risk of these low-income individuals to develop mental health problems [59], such as suicide attempt. However, a causal link between income and mental disorders cannot be drawn. Policymakers may need to consider interventions for mental disorders and suicidal behavior among low-income individuals.

Consistent with other studies, we found that women having a partner with controlling behaviour were more likely to attempt suicide [8] and to experience psychological distress as in [60], implying that coercive control is intimately relevant for the development of suicide risk in abused women. Our finding that women resident in rural areas were more likely to experience psychological distress is consistent with other studies [61]. This is a complex finding that warrants further research. Evidence from studies conducted in developed countries is contradictory about the elevated risk of suicide associated with rural residence. However, rurality as a risk factor has been explained in terms of socioeconomic stressors associated with the unpredictability of earning a living from farming and access to means (particularly firearms) [32].

### 7.1. Sensitivity Analysis

Comorbidity with psychological distress was associated with significantly elevated (ninefold) risk of suicide attempt, and vice versa, after controlling for sociodemographic factors. Similar findings have been found in developed countries [62], which found mental illness to increase the risk of suicide by a factor of 10 or more. Our findings therefore suggest that suicide attempts and psychological distress are highly comorbid, a point that has previously been made [63]. Thus it is possible that these disorders are significant risk factors for the other. For example, studies have found psychological distress to have the largest independent contribution to the risk of suicidal behaviour both in low- and middle-income countries as they are in high-income countries [64].

Several strengths of this study that are worth mentioning include the use of nationally representative data, adequate sample size, and use of comprehensive control of potential confounders (i.e., age, education, current working status, marital status, wealth index, place of residence, and controlling behaviour) in explaining association between intimate partner abuse and mental health outcomes. Several important limitations should be borne in mind when interpreting these results. The cross-sectional nature of the data is limited by retrospective recall biases and difficulty with understanding the temporal nature of the relationship between variables. It is theoretically possible that the mental health problems reported by the women could have predisposed them to experiencing intimate partner abuse. Data were based on retrospective self-report of the occurrence of suicidal behaviours and may be subject to underreporting and recall bias. Several factors are known to lead to underreporting of abuse and mental health consequences, including: (i) social desirability and the culture of silence [65], as respondents who highly desire social desirability have a stronger desire to be viewed positively and are more likely to underreport IPV incidents, particularly when information is collected via in-person interviews. Face-to-face reporting of socially undesirable abusive behaviours may evoke shame, guilt, and embarrassment, which possibly lower the likelihood of disclosure of such violence [66]; (ii) dependence, as women who are more economically dependent on their abusive husband/partner have a tendency to underreport partner abuse [67]; (iii) culture-specific factors that may be an obstacle that increases the resistance of perpetrators to report their violence [67]. DHS did not collect information from third-party informants to validate respondent reports. However, several systematic reviews have demonstrated that adults are capable of recalling past experiences with sufficient accuracy to provide valuable information [68]. The outcome mental disorders were not assessed using a comprehensive assessment of mental disorders in the DHS survey that are not specifically designed for making Diagnostic and Statistical Manual of Mental Disorders- (DSM-) based diagnoses. Future research on this topic should include the adequate measurement of these disorders.

Despite these limitations, this is the first study, to our knowledge, to examine and find a strong association between economic, physical, and psychological abuse and suicide attempts and psychological distress, as well as the greater predictability of psychological distress by economic abuse compared to either physical or psychological abuse.

## Figures and Tables

**Table 1:** Demographic and socioeconomic characteristics of the sample.

Characteristics	Suicide attempt			Psychological distress		
	No	Yes	Bivariate analyses	No	Yes	Bivariate analyses
	*N* ^†^ (%)	*N* ^‡^ (%)	OR (95% CI)	*N* ^*ψ*^ (%)	*N* ^§^ (%)	OR (95% CI)
Women's age (groups)	*P* = 0.440			*P* = 0.644		
15–19	185 (8)	12 (6)	0.52 (0.26–1.05)	103 (8)	94 (8)	0.88 (0.61–1.28)
20–24	333 (15)	24 (12)	0.58 (0.33–1.02)	186 (15)	171 (15)	0.89 (0.65–1.23)
25–29	413 (18)	36 (18)	0.70 (0.42–1.17)	252 (20)	196 (17)	0.75 (0.56–1.02)
30–34	421 (19)	37 (19)	0.71 (0.42–1.18)	244 (19)	214 (19)	0.85 (0.63–1.15)
35–39	371 (17)	31 (15)	0.67 (0.39–1.14)	209 (16)	193 (17)	0.90 (0.66–1.22)
40–44	279 (13)	29 (15)	0.83 (0.48–1.44)	156 (12)	152 (13)	0.94 (0.68–1.31)
45–49	233 (10)	29 (15)	1	129 (10)	133 (11)	1
Educational level	*P* < 0.01			*P* = 0.248		
No education	38 (2)	3 (1)	1.05 (0.32–3.46)	22 (2)	19 (2)	0.99 (0.54–1.86)
Primary	568 (25)	73 (37)	1.72 (1.26–2.33)	319 (25)	322 (28)	1.17 (0.97–1.40)
Secondary or higher	1629 (73)	122 (62)	1	938 (73)	812 (70)	1
Current working status	*P* < 0.01			*P* < 0.01		
Unemployed	1097 (49)	73 (37)	0.60 (0.45–0.82)	652 (51)	518 (45)	0.78 (0.67–0.92)
Employed	1138 (51)	125 (63)	1	627 (49)	635 (55)	1
Wealth index	*P* = 0.026			*P* < 0.01		
Poorest quintile	594 (27)	64 (32)	2.84 (1.44–5.63)	314 (24)	343 (30)	1.42 (1.07–1.89)
2nd poorest quintile	536 (24)	53 (27)	2.61 (1.31–5.21)	307 (24)	282 (24)	1.20 (0.90–1.60)
Middle quintile	476 (21)	44 (22)	2.44 (1.21–4.93)	302 (24)	218 (19)	0.94 (0.70–1.26)
2nd richer quintile	365 (16)	27 (14)	1.95 (0.93–4.10)	201 (16)	191 (17)	1.24 (0.91–1.69)
Richest quintile	264 (12)	10 (5)	1	155 (12)	119 (10)	1
Marital status	*P* < 0.01			*P* = 0.349		
Never/formerly married	282 (13)	12 (6)	1	151 (12)	143 (12)	1
Currently married	1953 (87)	186 (94)	2.24 (1.23–4.07)	1128 (88)	1010 (88)	0.94 (0.74–1.21)
Place of residence	*P* < 0.05			*P* < 0.01		
Urban	985 (44)	73 (37)	1	596 (47)	462 (40)	1
Rural	1250 (56)	125 (63)	1.35 (1.19–1.82)	683 (53)	691 (60)	1.30 (1.11–1.53)
Controlling behaviour	*P* < 0.001			*P* < 0.001		
No	714 (32)	33 (17)	1	460 (36)	286 (25)	1
Yes	1521 (68)	165 (83)	2.34 (1.59–3.44)	819 (64)	867 (75)	1.71 (1.43–2.04)
Justify wife beating	*P* = 0.493			*P* = 0.414		
No	1730 (77)	154 (78)	1	993 (78)	890 (77)	1
Yes	505 (23)	44 (22)	0.98 (0.69–1.39)	286 (22)	263 (23)	1.03 (0.85–1.24)

Note: OR = odds ratio; CI = confidence interval; % = percentage.

^*^ *P* < 0.05. ^**^ *P* < 0.01. ^***^ *P* < 0.001.

^†^ *N* = 2235; ^‡^ *N* = 198; ^*ψ*^ *N* = 1279; ^§^ *N* = 1153.

**Table 2:** Distribution and association of the outcome variables by the main exposure variables.

Characteristics	Suicide attempt			Psychological distress		
	No	Yes		No	Yes	
	*N* = 2235	*N* = 198		*N* = 1279	*N* = 1153	
	*n* (%)	*n* (%)	OR (95% CI)	*n* (%)	*n* (%)	OR (95% CI)
*Economic abuse *						
Not allowed to engage in legitimate work	∗∗∗			∗		
No	1783 (80)	143 (72)	1	990 (77)	936 (81)	1
Yes	452 (20)	55 (28)	1.52 (1.09–2.10)	289 (23)	217 (19)	0.79 (0.65–0.97)
Spouse controlled money or forced her to work	∗∗∗			∗∗∗		
No	2133 (95)	163 (82)	1	1240 (97)	1055 (91)	1
Yes	102 (5)	35 (18)	4.49 (2.96–6.80)	39 (3)	98 (9)	2.95 (2.02–4.32)
Destroyed personal property/pet or threaten to harm pet	∗∗∗			∗∗∗		
No	2077 (93)	157 (79)	1	1221 (95)	1012 (88)	1
Yes	158 (7)	41 (21)	3.43 (2.35–5.02)	58 (5)	141 (12)	2.93 (2.14–4.03)
Ever lost job/source of income because of husband	∗∗∗			∗∗∗		
No	2112 (94)	168 (85)	1	1229 (96)	1051 (91)	1
Yes	123 (6)	30 (15)	3.01 (1.95–4.65)	50 (4)	102 (9)	2.34 (1.65–3.32)
*Physical abuse *	∗∗∗			∗∗∗		
No	1778 (79)	105 (53)	1	1088 (85)	794 (69)	1
Yes	457 (21)	93 (47)	3.45 (2.57–4.65)	191 (15)	359 (31)	2.59 (2.12–3.15)
*Psychological abuse *	∗∗∗			∗∗∗		
No	1779 (80)	102 (51)	1	1088 (85)	792 (69)	1
Yes	456 (20)	96 (49)	3.67 (2.73–4.94)	191 (15)	361 (31)	2.60 (2.13–3.16)

Note: OR = odds ratio; CI = confidence interval; % = percentage.

^*^ *P* < 0.05. ^**^ *P* < 0.01. ^***^ *P* < 0.001.

**Table 3:** Multivariate analyses associations between economic, physical, and psychological abuse and suicide attempt and psychosocial stress.

Model variables	Suicide attempt			Psychosocial stress		
	Model 1^*ψ*^	Model 2^§^	Model 3^‡^	Model 1^*ψ*^	Model 2^§^	Model 3^‡^
	Crude OR (95% CI)	Adjusted OR (95% CI)	Adjusted OR (95% CI)	Crude OR (95% CI)	Adjusted OR (95% CI)	Adjusted OR (95% CI)
*Economic abuse (individual variables) *						
Not allowed to engage in legitimate work						
No	1	1	1	1	1	1
Yes	1.52 (1.09–2.10)	1.14 (0.79–1.63)	1.14 (0.79–1.63)	0.79 (0.65–0.97)	0.65 (0.53–0.81)	0.65 (0.52–0.81)
Spouse controlled money or forced her to work						
No	1	1	1	1	1	1
Yes	4.49 (2.96–6.80)	1.84 (1.13–2.99)	1.74 (1.06–2.86)	2.95 (2.02–4.32)	1.59 (1.04–2.43)	1.49 (0.97–2.29)
Destroyed personal property/pet or threaten to harm pet						
No	1	1	1	1	1	1
Yes	3.43 (2.35–5.02)	1.44 (0.93–2.25)	1.31 (0.84–2.06)	2.93 (2.14–4.03)	1.69 (1.19–2.40)	1.59 (1.12–2.26)
Ever lost job/source of income because of husband						
No	1	1	1	1	1	1
Yes	3.01 (1.95–4.65)	2.00 (1.24–3.23)	1.91 (1.18–3.08)	2.34 (1.65–3.32)	2.16 (1.49–3.13)	2.15 (1.47–3.13)
*Physical IPV *						
No	1	1	1	1	1	1
Yes	3.45 (2.57–4.65)	1.97 (1.39–2.80)	1.91 (1.34–2.71)	2.59 (2.12–3.15)	1.80 (1.45–2.24)	1.81 (1.45–2.26)
*Psychological IPV *						
No	1	1	1	1	1	1
Yes	3.67 (2.73–4.94)	2.19 (1.54–3.11)	2.02 (1.42–2.88)	2.60 (2.13–3.16)	1.84 (1.47–2.29)	1.75 (1.40–2.19)
*Educational level *						—
No education			0.69 (0.19–2.46)			
Primary			1.39 (0.97–1.98)			
Secondary or higher			1			
*Current working status *						
Unemployed			0.63 (0.46–0.86)			0.81 (0.68–0.96)
Employed			1			1
*Wealth index *						
Poorest quintile			1.98 (0.93–4.22)			1.15 (0.83–1.58)
2nd poorest quintile			2.15 (1.03–4.48)			1.06 (0.78–1.45)
Middle quintile			2.21 (1.06–4.62)			0.89 (0.65–1.21)
2nd richest quintile			1.81 (0.84–3.92)			1.19 (0.86–1.65)
Richest quintile			1			1
*Marital status *						—
Never/formerly married			1			
Currently married			1.54 (0.81–2.95)			
*Place of residence *						
Urban			1	1		1
Rural			1.24 (0.88–1.73)			1.32 (1.09–1.58)
*Controlling behaviour *				1		
Yes			1.66 (1.11–2.49)			1.43 (1.19–1.72)
No			1			1

Note: OR: odds ratio; CI: confidence interval; ^*ψ*^crude associations between outcomes and main exposure variables; ^§^adjusted associations between outcomes and main exposure variables; ^‡^adjusted associations including sociodemographic confounders.
